# Flame Spray Pyrolysis Synthesis of Vo-Rich Nano-SrTiO_3-x_

**DOI:** 10.3390/nano14040346

**Published:** 2024-02-11

**Authors:** Areti Zindrou, Pavlos Psathas, Yiannis Deligiannakis

**Affiliations:** Laboratory of Physical Chemistry of Materials & Environment, Department of Physics, University of Ioannina, 45110 Ioannina, Greece; a.zindrou@uoi.gr (A.Z.); pavlospsatha@gmail.com (P.P.)

**Keywords:** SrTiO_3_, strontium titanate, perovskite, flame spay pyrolysis, oxygen vacancies, Vo, lattice defects, photocatalyst

## Abstract

Engineering of oxygen vacancies (Vo) in nanomaterials allows diligent control of their physicochemical properties. SrTiO_3_ possesses the typical ABO_3_ structure and has attracted considerable attention among the titanates due to its chemical stability and its high conduction band energy. This has resulted in its extensive use in photocatalytic energy-related processes, among others. Herein, we introduce the use of Flame Spray Pyrolysis (FSP); an industrial and scalable process to produce Vo-rich SrTiO_3_ perovskites. We present two types of Anoxic Flame Spray Pyrolysis (A-FSP) technologies using CH_4_ gas as a reducing source: Radial A-FSP (RA-FSP); and Axial A-FSP (AA-FSP). These are used for the control engineering of oxygen vacancies in the SrTiO_3-x_ nanolattice. Based on X-ray photoelectron spectroscopy, Raman and thermogravimetry-differential thermal analysis, we discuss the role and the amount of the Vos in the so-produced nano-SrTiO_3-x_, correlating the properties of the nanolattice and energy-band structure of the SrTiO_3-x_. The present work further corroborates the versatility of FSP as a synthetic process and the potential future application of this process to engineer photocatalysts with oxygen vacancies in quantities that can be measured in kilograms.

## 1. Introduction

Perovskite-oxides with the classical ABO_3_ structure have attracted considerable attention for their functional properties, e.g., their multiferroic properties [1,2]. They are used in the electronics industry [3], in enhanced power conversion efficiencies [4]; and they are also used extensively as photocatalysts due to their structural, compositional and stoichiometric flexibility [5]. Among the titanates, much attention has been paid to strontium titanate (SrTiO_3_), a perovskite with a cubic structure [6] that possesses several advantageous features, including low cost [7], chemical stability and thermal resilience (melting point reaching 2080 °C); carbon and sulfur tolerance further contributes to the structural stability [8]. Its highly-reducing conduction-band-edge energy position (E_CB_) at −1.2 eV vs. NHE (Normal Hydrogen Electrode) [9,10] renders SrTiO_3_ as a promising platform for H_2_-production or CO_2_-reduction photocatalytic systems. However, SrTiO_3_ is characterized by a drawback; namely, a wide 3.2 eV band gap, restricting absorption to ultraviolet (UV) photons [11]. To overcome this constraint, a variety of strategies are implemented. For instance, structural defect engineering (metal or oxygen vacancies), element doping and co-catalyst heterostructures have been implemented, with great results [12].

SrTiO_3_ has demonstrated high optimizability for specific technologies through crystal lattice engineering, enabling the control of structural, optical, and electronic properties to enhance photocatalytic efficiency [13,14]. In this context, oxygen vacancies (Vo) exert a remarkably influential role in various optoelectronic properties [12].

Vo-engineering allows fine-tuning of various properties; e.g., surface Vos can act as photogenerated-species’ traps, facilitating the transfer to adsorbed compounds and consequently averting {electron-hole, e^−^/h^+^} recombination. In contrast, typically, bulk Vos can merely function as {e^−^} traps, facilitating the recombination of photogenerated {e^−^/h^+^}; i.e., diminishing photocatalytic performance [15]. Recently, we have exemplified this for the case of Vo-rich ZrO_2-x_ [16] where diligent control of the amount and location of Vo was the key parameter for enhancing its photocatalytic H_2_-production.

Numerous efforts have been reported in the literature regarding the introduction of oxygen vacancies in the SrTiO_3_ lattice. Recently, Li et al. have reported that the formation of oxygen vacancies in SrTiO_3_ nanofibers through calcination in an H_2_/N_2_ atmosphere can significantly improve photocatalytic H_2_ production by facilitating charge transfer and slowing down their recombination without the use of a cocatalyst [17]. Similar results have been reported by Fan et al.; heating of pristine SrTiO_3_ under a carbon-containing reducing atmosphere yielded a distorted surface layer with oxygen vacancies [18]. These vacancies can provide suitable energy levels for visible light activity and improve charge separation. In another work, Qin et al. have demonstrated that the reduction of oxygen vacancies in SrTiO_3_ by introducing La^3+^ and Al^3+^ qualifies as an effective strategy to boost photocatalytic H_2_ and O_2_ evolution [19]. Doping with Al^3+^ introduces the oxygen vacancies into the perovskite, resulting in a conversion of Ti^3+^ to Ti^4+^. At the same time, La^3+^ doping balances the effect of Al^3+^, reversing the transformation trend of the semiconductor. However, a common problem among various synthetic approaches is the milligram-scale quantities they yield and the size of the produced nanoparticles, which exceeds 200 nm. 

In this context, flame spray pyrolysis (FSP) is a versatile technology for the engineering of multifunctional nanostructures and nanodevices [20] with controllable characteristics (size, phase, crystallinity), and can provide nanoparticles at large quantities. Recently, we have demonstrated that FSP can be successfully employed to synthesize highly-photoactive perovskite materials, e.g., BiFeO_3_ [21], NaTaO_3_ [22]. Herein, we show that FSP-made SrTiO_3_ with controlled Vos is a novel approach towards enhanced photoactivity. Previously, Yuan et al. presented FSP-made SrTiO_3_ with dopings of Co, Fe, Mn, Ni and Cu [23,24]. Our previous work showcased La-doping and CuO-heterojunction on SrTiO_3_ enhancement and selective H_2_ vs. CH_4_ photocatalytic production from H_2_O/CH_3_OH [25].

Herein, the specific aims of the present works were: (i) to develop FSP protocols for controlled Vo-engineering in nanosized SrTiO_3-x_, employing an advanced FSP approach in which the combustion stoichiometry was controlled by the FSP-design and operating conditions [16,20,26,27]; (ii) to study the structural and spectroscopic properties of the so-produced SrTiO_3-x_; (iii) to extend and highlight the versatility of A-FSP as a technique capable of producing large quantities of elaborated nanostructures. 

## 2. Materials and Methods

### 2.1. Synthesis of Reduced SrTiO_3-x_ by Anoxic Flame Spray Pyrolysis (A-FSP)

**Figure 1:**
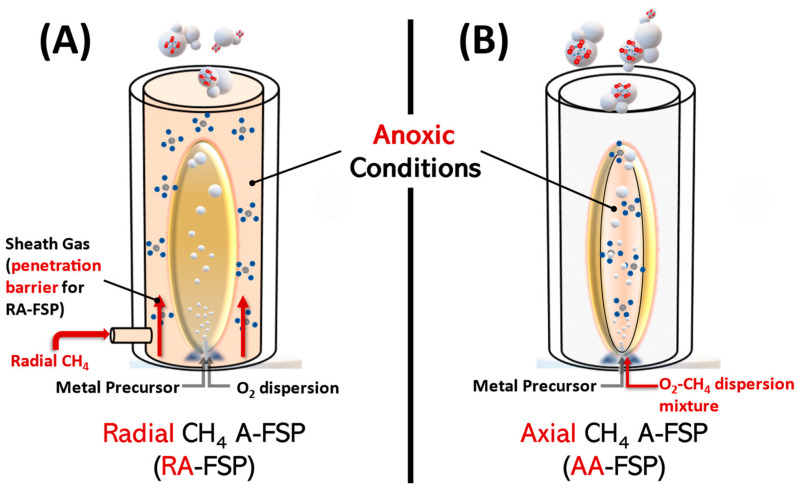
(**A**) Schematic representation of Radial-CH_4_ and (**B**) Axial-CH_4_ A-FSP processes.

A library of four SrTiO_3-x_ (for convenience, these are codenamed STO) perovskites was prepared in an enclosed single-nozzle FSP reactor using two different Anoxic-FSP configurations (see Figure 1A,B). The A-FSP reactor set-up uses CH_4_ to control the anoxic-combustion environments, as exemplified in our recent works [16,26,27]. This CH_4_ is additional to the classically used CH_4_ in the pilot-flame of FSP [28]. The two A-FSP configurations in Figure 1A,B are codenamed Radial- and Axial-A-FSP; these define the way in which CH_4_ is introduced, respectively. In all cases, the FSP-nozzle was enclosed by a cylindrical metal chamber consisting of two concentric tubes, a sinter metal tube (outer tube) and a perforated metal tube (inner tube) to isolate the flame from the surrounding atmosphere, as has been described in detail in previous works [16,26,27,29]. The so-produced materials (listed in Table 1) are codenamed STO-R and STO-A for Radial- and Axial-A-FSP, respectively. 

Precursor solution was prepared by dissolving 0.2M Strontium acetate (97%, STREM (Newburyport, MA, USA)) and 0.2M Titanium (VI) isopropoxide (97%, Sigma-Aldrich (Saint Louis, MO, USA)) in a 1:1 mixture of acetic acid and xylene for the synthesis of perovskite SrTiO_3_. The solution was fed into the FSP burner through a capillary at a flow rate of P = 5 mL min^−1^ and atomized into fine droplets using a dispersion flow rate of D_total_ = 5 L min^−1^. The resulting spray was ignited and sustained by a premixed oxygen/methane pilot flame (O_2_ 4 L min^−1^, CH_4_ 2 L min^−1^) to initiate combustion. Finally, the pressure drop was fixed at 1.5–2 bars and the produced particles were collected in a glass microfiber filter (Hahnemühle GF 6 257) with the aid of a vacuum pump (BUSCH V40) and collected by scraping.

#### 2.1.1. Radial-CH_4_ Anoxic Flame Spray Pyrolysis (RA-FSP)

This RA-FSP process is similar to conventional FSP regarding the particle formation steps. Its difference lies in the radially introduced CH_4_ gas surrounding the flame (Figure 1A). In addition, a suitable low sheath gas (N_2_) flow of 5 L min^−1^ is used to allow the radially introduced CH_4_ to penetrate the sheath gas barrier and react with the flame and the particles. Note that the role of N_2_-sheath is double, maintaining the anoxic atmosphere during particle synthesis and aiding in the particle collection; i.e., via upwards convection. The production protocol was set up so that the materials are consistent with each other, i.e., precursor molarity, the pilot flame, and the P/D ratio were constant; thus, their comparison was focused on the additional insertion of CH_4_; see Table 1. The so-produced materials are listed in Table 1 and codenamed “STO-RX” where x = the radial CH_4_-inflow in L min^−1^.

#### 2.1.2. Axial CH_4_ Anoxic Flame Spray Pyrolysis (AA-FSP)

In AA-FSP, a mixture of oxygen (O_2_)/methane (CH_4_) is used as a dispersion gas to promote a reducing environment insitu inside the flame [16,26]. The high combustion enthalpy of methane, i.e., 50–55 MJ kg^−1^, increases the combustion temperature, which subsequently minimizes the deposition of graphitized carbon. In contrast to Radial A-FSP, it is employed at the moment of particle crystallization; thus, there is a higher chance of creating deficient centers in the material. By keeping constant the pilot flame and the P/D ratio and adjusting the D_1_ (O_2_)/D_2_ (CH_4_), two materials were prepared—codenamed STO-A1 and STO-A2—where D_2_ = 1 L min^−1^ and 2 L min^−1^, respectively. Herein, an N_2_ sheath gas flow of 10 L min^−1^ was also used to decrease the overall oxygen concentration during the synthesis. 

### 2.2. Structural Characterization of Materials

Powder X-ray diffraction (pXRD) was employed to analyze the crystal phase and structural properties of the flame-synthesized strontium titanate (STO) nanomaterials. A Bruker D8 Advance diffractometer employing CuKa radiation with a wavelength (λ) of 1.5405 Å, was utilized for the characterization. The scanning parameters included a step size of 0.03°, a scanning rate of 2 s per step, and a 2-theta angle range spanning from 10° to 80°. The diffractometer operated at a current of 40 mA and a generator voltage of 40 kV. The average crystallite size of the FSP-made nanopowders was calculated using the Scherrer equation [30] (1).

(1) $$d_{XRD}=\frac{K\lambda }{\left(FWHM\right)\times cos\theta }$$

where *d*_XRD_ is the crystallite size in nanometers (nm), K is a shape constant (0.9 in our case), λ is the wavelength of CuKa radiation, FWHM is the full width at half-maximum of the XRD peaks and θ is the peak diffraction angle. Further analysis of the XRD data involves Rietveld refinement using Profex, a graphical user interface program, which can yield the lattice parameter *α*. 

Transmission Electron Microscopy (TEM) was utilized to investigate the morphology of the materials. This examination was carried out using an FEI Titan 80–300 S/TEM microscope, operating at a 300 kV accelerating voltage and a 21.5 mrad beam convergence angle. Prior to measurement, each nanopowder was dispersed in ethanol and subjected to sonication in a bath sonicator. The resulting suspension was then deposited as a single droplet onto a copper TEM grid coated with a thin carbon layer. To eliminate potential organic contaminants, the samples underwent a 3-s treatment in argon plasma using a Fischione Instruments 1020 Plasma Cleaner.

Ultraviolet-Visible Diffuse-Reflectance (UV-Vis DRS) absorption spectra were recorded using a PerkinElmer (Lambda 35) spectrometer to ascertain the energy gap values. BaSO_4_ powder served as the standard background. The spectra were obtained at room temperature within the 200–800 nm range, employing a scanning step of 1 nm. The Kubelka–Munk method was then applied to calculate the energy gap values (Eg) [31]. 

Brunauer–Emmett–Teller (BET) analysis was employed to measure the Specific Surface Area (SSA) and the pore size distribution of the nanomaterials. A Quantachrome NOVAtouch_LX2 instrument was used to record the N_2_ adsorption–desorption isotherms at 77 K. Prior to measurement, at least 200 mg of the FSP-made nanopowder was degassed at 100 °C for 16 h. The SSA values were calculated using the absorption data points in the range of 0.1−0.3 relative pressure P/Po. Pore radius analysis was obtained by the Barett–Joyner–Halenda (BJH) method [32] in the range of 0.35–0.99 P/Po. Moreover, based on BET data we can calculate the average particle diameter (*d*_BET_) in nanometers using the following equation:

(2) dBET=6000SSABET in m2g×(ρ in gcm3)

where ρ symbolizes the density; in the case of SrTiO_3_ this is equal to 5.11 g cm^−3^. However, there are limitations in this calculation, i.e., the particles must be spherical or quasi-spherical.

Raman spectra were recorded using a HORIBA-Xplora Plus spectrometer coupled with an Olympus BX41 microscope. As an excitation source, a 785 nm diode laser was used and with the aid of a microscope the beam was focused on the sample. Before each measurement, each powder material was gently pressed between two glass plates to form a pellet-like shape. Raman spectra were recorded in the range of 100–1700 cm^−1^ and the spectra resolution was approximately 1–1.5 cm^−1^, performing 30 accumulations at fixed intensity, i.e., 50% of the total intensity of the laser.

X-ray photoelectron spectroscopy (XPS) was utilized to investigate the oxidation states of Sr, Ti and O atoms. This analysis was conducted with a SPECS spectrometer featuring a twin Al-Mg anode X-ray source and a multi-channel hemispherical sector electron analyzer (HSA-Phoibos 100, Mansfield, MA, USA). The XPS measurements utilized a monochromatized Mg Kα line at 1253.6 eV, an analyzer pass-energy of 15 eV, and a base pressure of 2–5 × 10^−9^ mbar. Binding energies were referenced to the energy of the C1s carbon peak at 284.5 eV. Peak deconvolution was carried out using mixed Gaussian–Lorentzian functions, employing WinSpec software developed at the Laboratoire Interdisciplinaire de Spectroscopie Electronique, University of Namur, Belgium.

Thermogravimetry-differential thermal analysis (TG-DTA) was conducted to determine the mass change (Δm) of the nanopowders using a Setaram Labsys Evo instrument using a heat rate of 2 °C min^−1^ from 20 to 700 °C and a flow of synthetic air gas of 20 mL min^−1^.

## 3. Results

**Figure 2:**
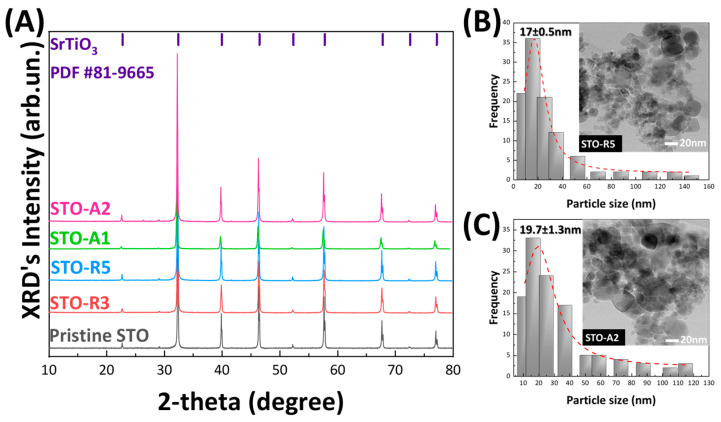
(**A**) XRD patterns of the pristine and reduced STO nanomaterials. (**B**,**C**) Size distribution graphs obtained from several TEM images for STO-R5 and STO-A2 materials. (*Inset Figures:* TEM image of STO-R5 and STO-A2).

Figure 2A shows the XRD patterns of the as-prepared nanomaterials, where the diffraction peaks match the diffraction peaks of the cubic perovskite structure of SrTiO_3_ (PDF #81-9665). However, a closer inspection of the XRD patterns reveals the presence of small peaks at 26.4°, 29.1°, 35.8°, 41.7°, 51.8° and 60.4° that do not match the known XRD patterns of TiO_2_ and/or SrO. Therefore, we attribute these small peaks to the presence of minor impurities. Thus, the XRD data confirm that both RA-FSP and AA-FSP protocols produce highly crystallized SrTiO_3_ nanoparticles. *d*_XRD_ values were calculated from the peak at 32.4°, which corresponds to (110) using the Scherrer formula [30] and range between 41–58 nm, listed in Table 2.

TEM images (Figure 2B,C) show the formation of quasi-spherical SrTiO_3_ particles forming neck-sintered aggregates, which are typical for FSP-made particles. Analysis of the TEM images using ImageJ, an open-access program, reveals the existence of a size-distribution from 10 to 150 nm (see Figure 2B,C inset figures), with most of the particles being between 10 and 40 nm, with a mean size of *d*_TEM_ = 17 ± 0.5 nm and 19.7 ± 1.3 nm for STO-R5 and STO-A2, respectively. We underline that these size distributions are a result of at least 100 particles and were obtained from several TEM images. These results are in good agreement with the structural characterization results of FSP-made SrTiO_3_ particles from our previous work [25]. Comparison of *d*_XRD_ and *d*_TEM_ reveals a well-known effect in which larger particles dominate the diffraction patterns, while the contribution of smaller particles is less visible. Thus, XRD tends to overestimate the average particle size, while TEM gives us better information regarding the morphology of the material. 

The N_2_ adsorption/desorption isotherms (see Figure S1A–E) show the characteristic type-III isotherm with a negligible pore volume. A careful examination of the SSA values, listed in Table 2, and comparison with the XRD results indicates that: (i) *d*_XRD_ shows a moderate increase as CH_4_-flows become higher, i.e., STO-R5 or STO-A2; and (ii) *d*_BET_ dramatically increases in STO-A1, STO-A2 (Table 2), i.e., when the Axial-A-FSP set-up was used. This *d*_BET_ increase was less-prominent in the STO-R3, R5 particles. This increase in *d*_BET_ indicates a strong promotion of the particle-aggregation that occurs when CH_4_ is introduced in the dispersion-gas flow, which decreases when we enclose the burner and introduce CH_4_ to the FSP set-up. These trends can be understood as follows: in the FSP process, high temperature residence time is a key characteristic of the flame synthesis, which determines particle growth [33]. In an enclosed FSP-flame, introduction of CH_4_—either radially or axially—increases the combustion enthalpy, resulting in higher temperatures that drive the growth of particle size.

**Figure 3:**
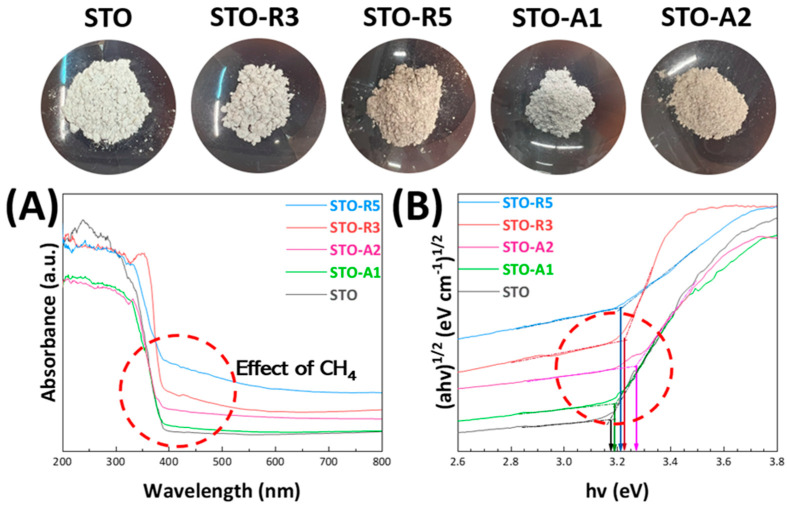
(**A**) UV−Vis DRS absorption spectra of STO nanomaterials. (**B**) Tauc plots with the arrows pointing at the calculated Eg values of the STO nanomaterials. (*Top Figures:* Powder nanoparticles showcasing the color change).

The optical properties were measured using UV-Vis DRS spectroscopy. Pristine STO as a semiconductor possesses an indirect energy gap (E_g_) of 3.25 eV and a direct energy gap of 3.75 eV. The bandgap values were estimated with a Tauc plot using the Kubelka–Munk method [31]; see Figure 3B. As shown in Figure 3A, all FSP-produced materials exhibit an absorption edge that begins at 390 nm, yielding an energy gap of 3.2 eV approximately. For convenience, the E_g_-values of the materials are listed in Table 2. Although the E_g_ values seem not to be affected by much, i.e., E_g_ ~ 3.1–3.2 eV, a strong offset is observed in the UV-Vis DRS spectrum of all anoxic STO materials, with most prominent that for STO-R5 > STO-R3 > STO-A2 > STO-A1. This trend, together with minor shifts in the E_g_ values, is interpreted as being manifestations of the formation of Urbach-states [34]. In brief, Urbach-states appear as band-tails at the edges of the conduction- and valence-band in semiconductors, and can typically be created in partially reduced oxides [35]. Due to their quasi-symmetric distribution, these band-tails do not manifest themselves in clear-change in the band-gap, but they rather cause characteristic distribution of the UV-Vis absorbance extending over a wide range of wavelengths; thus, there is an upshift in the absorbance profile, as shown in Figure 3. This reveals that in the FSP process CH_4_ creates a distribution of density-of-states at the edges of the conduction band (CB) and valence band (VB) of the STO, with a more profound effect of Axial-CH_4_; i.e., see the extreme case of STO-R5. We underline that in the case of Urbach-tails the estimation of the E_g_-values should be done correctly, according to the method of Makula et al. [36]. In this method, the upshifted baseline should be taken to estimate the crossing point with the tangential-slope typically used in the Tauc analysis; see Figure 3B. 

It is instructive to connect the effect of these Urbach-states with the drastic effect on the color of anoxic material, as shown in the photos in Figure 3 (top). The initially crisp white color of STO turned into beige-grey, without having a profound effect on the E_g_ values. 

**Figure 4:**
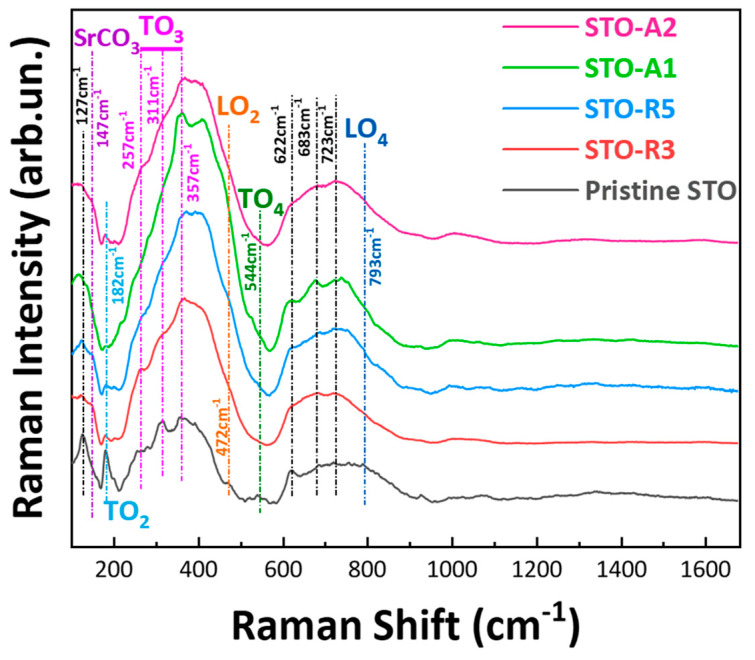
Raman spectra of the FSP−made STO nanomaterials, with the characteristic transverse optical band modes of TO_1_, TO_2_, TO_3_, and TO_4_ and, additionally, the characteristic longitudinal optical band modes of LO_2_ and LO_4_.

To get more insight into the atomic-level effect of the anoxicity on the STO-lattice, we used Raman spectroscopy to study the STO-lattice dynamics because of its sensitivity to identify short-range distortions, structural phase transitions and defects introduced in the lattice. At room temperature, STO possesses an ideal cubic perovskite structure in which first-order Raman scattering is forbidden. As Petzelt et al. [37] have reported, when symmetry is broken—either by strain engineering created from lattice mismatch or by surface reconstruction and relaxation phenomena—first-order Raman scattering can be observed. Figure 4 shows the Raman spectra for the A-FSP-made STO nanomaterials. There are two primary modes in the spectrum where TO stands for transverse optical and LO for longitudinal optical branches assigned as follows: TO_2_ (182 cm^−1^), TO_3_ (257–357 cm^−1^), LO_2_ (472 cm^−1^), TO_4_ (544 cm^−1^), broad band around 622–723 cm^−1^ and LO_4_ (793 cm^−1^). We also observe a small intensity peak at 147 cm^−1^ which corresponds to SrCO_3_ [38] and could be formed during FSP synthesis. However, XRD data (Figure 2A) do not reveal the presence of SrCO_3_, thus we could conclude that SrCO_3_ is present in the materials in a small amount, possibly located on the surface. An alternative interpretation was given by Deltreggia et al., in which the peak at 149 cm^−1^ was assigned to TO_1_ mode [39]. The peak at 147–149 cm^−1^ may contain contributions from to TO_1_ mode of SrTiO_3_ or the SrCO_3_ phase, i.e., according to Deltreggia et al. However, the SrCO_3_ phase should be strongly evidenced by a peak at 1074 cm^−1^. This is absent in our Raman data. Thus, we exclude assignment to SrCO_3._ The absence of this phase is also evidenced from the XRD. The above-mentioned phonon branches and their respective values are summarized in Table 3. The lowest frequency modes (LO_1_) arise due to the B-ion motion against oxygen vibrations. The intermediate frequency modes (LO_2_/TO_2_) emerge from A-ion vibrations and the highest frequency vibrations are due to oxygen vibrations in BO_6_ octahedra in the ABO_3_ lattice.

The presence of first-order Raman peaks at 182 cm^−1^ (O-Sr-O), 257 cm^−1^ (O-Sr-O) and 544 cm^−1^ (Ti-O-Ti) indicates that FSP-made STO perovskite is not strain-free. This is understood if we consider that FSP synthesis is accomplished in a matter of seconds. In this time window the ‘A’ atoms, which are usually larger than the ‘B’ atoms, will form a crystal. Du et al. [40] have also observed the first-order modes in polycrystalline SrTiO_3_ even at 300 K, which is attributed to oxygen vacancies and strain effects. Moreover, the presence of dopants, e.g., such as Ca, or the application of an external electric field was found to break the symmetry of SrTiO_3_; hence, this causes the appearance of first-order phonon modes [41,42]. 

**Table 3:** Summarized Raman band frequencies and their corresponding phonon branches of SrTiO_3_.

Phonon Branch	Assignment	Raman Shift (cm^−1^)	Raman Shift (cm^−1^) (Literature)
TO_1_ or SrCO_3_	Ti-O-Ti or SrCO_3_	147	149 [38], 149 [39]
LO_1_, TO_2_	O-Sr-O	182	178 [38], 177 [39], 180 [43],190 [44]
TO_3_, LO_3_	O-Sr-O	257	271 [38], 289 [39], 274 [43], 250–348 [44]
		311	
		357	
LO_2_		472	482 [39]
TO_4_	Ti-O-Ti	544	543 [38], 545 [39], 546 [43], 539 [44]
		622	730 [39], (591, 655, 713) [43], 621–718 [44], (617, 667, 722) [45]
		683	
		723	
LO_4_	Ti-O	793	795 [38], 795 [39], 803 [43], 786 [44]

In this context, the present Raman data reveal that the use of CH_4_, either axially or radially, exerts a dramatic effect on TO_2_-modes. Theoretically, a TO_2_ mode is associated with vibrations of A-ion, herein the Sr, in the ABO_3_ structure and a potential increase is due to distortions in the order of a wavelength in the STO lattice [46]. Moreover, TO_2_ and TO_4_ are polar modes while TO_3_ is a non-polar mode that corresponds to the bending of the O_6_ octahedra [47]; variation of the polar modes points out the polarization characteristics of STO nanoparticles (NPs). Wu et al. [45] had observed an increase in the intensity of the TO_2_ mode, which was correlated with a decrease in the grain size. Thus, in the present cases the enhanced intensity of TO_2_ mode implies the formation of micropolar regions in STO NPs, which can be ascribed to the enhanced surface-defect dipoles on the grain boundaries [45]. Interestingly, TO_3_ mode exhibits different behavior than TO_2_. Previous studies have shown that TO_3_ phonon activation is associated with long-range structural distortions [48]. Therefore, the intensity increase of TO_3_ mode in STO-R3, STO-R5, STO-A1 and STO-A2 is interpreted as a suppression of long-range structural distortion [40,45]. Wu et al. have observed the softening of TO_2_ and TO_3_ modes by decreasing the grain size of SrTiO_3_, which suggests an increase of the Ti-O bond length. Their observation was coupled with an increase of the lattice parameter *α* from 3.907 Å to 3.922 Å when decreasing the size from 80 nm to 10 nm [45]. In our case, we observe a loss in the intensity of TO_2_ and the softening of TO_3_ modes, going from STO to the more reduced materials, i.e., STO-R5 and STO-A2. Rietveld analysis of the XRD pattern yields a cubic lattice parameter α = 3.906 Å, 3.905 Å and 3.906 Å for STO, STO-R5 and STO-A2, respectively; values that are close to that of the ideal STO (3.905 Å) [37]. Importantly, the FSP-made nano-STO’s observed loss of TO_2_ mode indicates an increased lattice-symmetry. This could be correlated with an increase in combustion-enthalpy, i.e., due to excess CH_4_. This implies that the role of CH_4_ in A-FSP technology might be more complex than simply creating a reducing environment. For FSP-made perovskites this increase in enthalpy could either affect the crystallinity of the final material or may result in materials with higher lattice symmetry. This beneficial role is evident in the Raman spectra through the loss of modes that signify a break of symmetry. 

**Figure 5:**
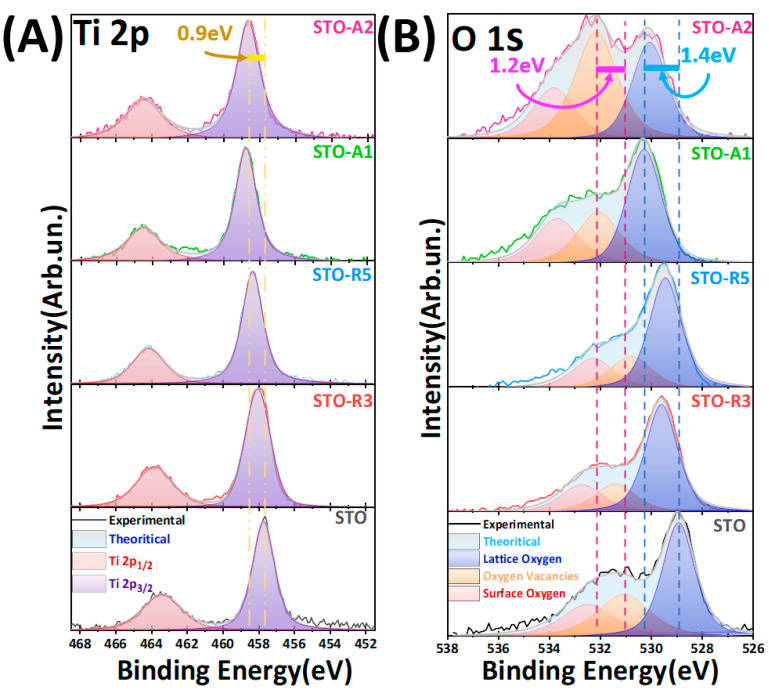
(**A**) XPS measurements of the five materials for the fitted Ti 2*p*_1/2_ and Ti 2*p*_3/2_ XPS spectra; (**B**) oxygen XPS measurements of the five materials for the fitted lattice oxygen, oxygen vacancies, and surface oxygen-XPS spectra.

To further investigate the effect of CH_4_ during the synthetic process, X-ray photoelectron spectroscopy (XPS) has been employed. Figure 5A,B present the spectra of Ti 2*p* and O 1*s*, respectively. Additional XPS data concerning the Sr 3*d* can be found in Supplementary Materials Figure S2. The primary focus is directed towards titanium and oxygen, with the aim of discerning oxygen vacancies and reduced states within the crystal structure of the five materials.

Figure 5A shows the Ti 2*p* XPS spectra, with the five materials possessing binding energies attributed to the Ti 2*p_3/2_* and Ti 2*p_1/2_* corroborating the oxidation state of Ti^4+^. The pristine material has, correspondingly, 457.7 eV and 463.4 eV [49], but with increased anoxic FSP conditions a gradual higher energy shift is observed on the XPS signals of Ti 2*p* and O 1*s*. The largest energy shift of 0.9 eV occurs with the axial CH_4_ conditions. The phenomenon of XPS shifting to higher binding energies has occurred with other synthesis methods, resulting in SrTiO_3_ that have oxygen vacancies [15,17] as well as TiO_2_ materials that have oxygen vacancies [50,51]. The axial or radial insertion of CH_4_ induces defects in the SrTiO_3_ crystal structure that push the Fermi level upwards by increasing the equilibrium electron density, resulting in the 0.9 eV positive shift [52,53]. Regarding the Strontium XPS data presented in Figure S2, the observed binding energies for the five materials were approximately 133.2 eV and 134.9 eV; which corresponds to Sr 3*d_5/2_* and Sr 3*d_3/2_*, respectively. These values denote the oxidation state of Strontium as the Sr^2+^ state [13,54].

For the O 1*s* XPS spectra, as depicted in Figure 5B, three Gaussian peaks have been carefully fitted. For the pristine SrTiO_3_ (STO) material, a binding energy of 529 eV is identified. This corresponds to O^2−^ ions inherent in the crystal lattice structure, denoting lattice oxygen species [13,15]. At 532.5 eV, a peak is attributed to loosely bound oxygen derived from adsorbed oxygen molecules on the particle surface or hydroxyl groups [13,15]. The intermediate peak at 531.1 eV is associated with the concentration of oxygen vacancies within the structure [55,56]. As it is evident from the O 1*s* XPS in Figure 5B, even the pristine FSP-made STO has a high percentage of oxygen vacancies. The introduction and increase of the Radial-CH_4_ causes a strong alteration in the oxygen vacancies population, shifting the oxygen species peaks. Unlike Radial-CH_4_, the axially introduced CH_4_ has a greater effect on the oxygen populations and species. The pronouncedly elevated area and intensity ratios in STO-A2 further support the abundance of inherent oxygen vacancies. 

To further study the materials and their mass change (Δm), TG-DTA measurements were performed (Figure S3). Based on the XPS measurements, STO-A1 and STO-A2 have been selected due to their higher amounts of Vos. We have divided the TGA profile in two regions of interest. First, we have the low-temperature region between 25 °C and 200 °C, which corresponds to the loss of physiosorbed water from the nanoparticles [57]. Second, in the region between 200 and 550 °C, we have mass loss due to the decomposition of C-O, C-C and C-H bonds of non-graphitized carbons and residual solvents [57]. We notice that pristine STO exhibits a mass loss of 6.5%, of which 2% is due to physiosorbed water and 4.5% is due to uncombusted solvents and carbon bonds, which is typical for FSP materials. However, STO-A1 and STO-A2 have a completely different TGA profile compared to STO. Both materials have a similar behavior in which their mass change is positive, i.e., gaining weight, and reaches a maximum at 250 °C of 1% and 2% for STO-A1 and STO-A2, respectively. Then we have a slight loss from 250 °C to 400 °C, and finally, from 400 °C to 700 °C their mass change is positive once again. Since the TGA measurements were performed under synthetic air, we can attribute this behavior of STO-A1 and STO-A2 to oxygen uptake filling the Vos that were created during A-FSP synthesis. 

Regarding the FSP process, we notice that Axial-CH_4_ in which less CH_4_ is applied has a greater impact on oxygen vacancies formation in the materials vs. the Radial-CH_4_ in which higher CH_4_-flows prevail. This can be understood if we take into account that in AA-FSP the CH_4_ is introduced inside the flame; thus, it affects the primary particles created in the first stages of particle formation in FSP. On the other hand, Radial-CH_4_ has a more subtle effect on the final material, creating fewer oxygen vacancies since the CH_4_ affects the STO particles after their formation. Overall, the present results exemplify the ability of A-FSP to control at a fine level the vacancies and lattice properties of perovskite SrTiO_3_.

## 4. Conclusions

In the present work, we extend the versatility of the Anoxic-FSP synthesis to the family of SrTiO_3_ perovskite materials. We exemplify an industrial-synthesis method that allows fine control of the lattice microstructure and vacancies. Raman spectroscopy reveals the existence of long-range structural distortions on the Ti-O bond length. Moreover, the appearance of first-order Raman scattering in the majority of the materials hints at the presence of strain effects and oxygen vacancies. These results are further supported by XPS measurements. Axial- and Radial-CH_4_ induces large energy shifts, increasing the equilibrium electron density. O 1*s* XPS spectra show that Axial-CH_4_ can have a greater effect on the formation of oxygen vacancies than Radial-CH_4_. TGA analysis reveals that while pristine STO exhibits the expected profile for FSP-made nanoparticles, the reduced STO-A1 and STO-A2 have a totally different profile, and gain weight; this further proves the existence of Vos. The modified dispersion feed used in the case of Axial-CH_4_ creates a highly reducing environment at the heart of the flame affecting the particles in the early stages of their creation. Technology-wise, the present findings could provide new insights into the large-scale synthesis of reduced perovskites from FSP technology. 

## Figures and Tables

**Table 1:** A-FSP conditions for the synthesis of Vo-rich SrTiO_3-x_ perovskites.

Nanomaterial	Sheath Gas (L min^−1^)	P/D	Axial CH_4_ (L min^−1^)	Radial CH_4_ (L min^−1^)
Pristine STO	O_2_: 10	5/5	-	-
STO-R3	N_2_: 5	»	-	3
STO-R5	N_2_: 5	»	-	5
STO-A1	N_2_: 10	»	1	-
STO-A2	N_2_: 10	»	2	-

**Table 2:** Structural characteristics of the reduced STO nanoparticles.

Nanomaterial	*d*_XRD_ (nm)	*d*_BET_ (nm)	SSA (m^2^ g^−1^) (±0.5)	Band Gap (E_g_) (eV) (±0.05)
Pristine STO	45 ± 0.5	36 ± 0.5	32.3	3.17
STO-R3	41 ± 0.5	53 ± 0.5	22.2	3.23
STO-R5	58 ± 0.5	68 ± 0.5	17.4	3.22
STO-A1	43 ± 0.5	110 ± 0.5	10.7	3.19
STO-A2	54 ± 0.5	96 ± 0.5	12.2	3.27

## Data Availability

Data are contained within the article and supplementary materials.

## Supplementary Materials

The following supporting information can be downloaded at: https://www.mdpi.com/article/10.3390/nano14040346/s1, Figure S1. (A–E) N2 absorption-desorption isotherms of Pristine-STO, STO-R3, STO-R5, STO-A1 and STO-A2 perovskites synthesized using the A-FSP process. Inset: Pore size distribution plot using the BJH method. Figure S2. (A–E) Sr 3*d* XPS spectra of the five FSP and A-FSP-made STO nanomaterials. Figure S3. TGA data of the materials Pristine STO, STO-A1 and STO-A2 under synthetic air. Vertical arrows were used to mark the estimated mass change (Δm).
