# Supplementary material for: Flame Spray Pyrolysis Synthesis of Vo-Rich Nano-SrTiO3-x
Source: Nanomaterials (Basel). 2024 Feb 11;14(4):346. doi: 10.3390/nano14040346 (PMC10891825; doi:10.3390/nano14040346)
Supplement: Supplementary file 1 [file nanomaterials-14-00346-s001.zip › nanomaterials-2810466-supplementary.pdf]

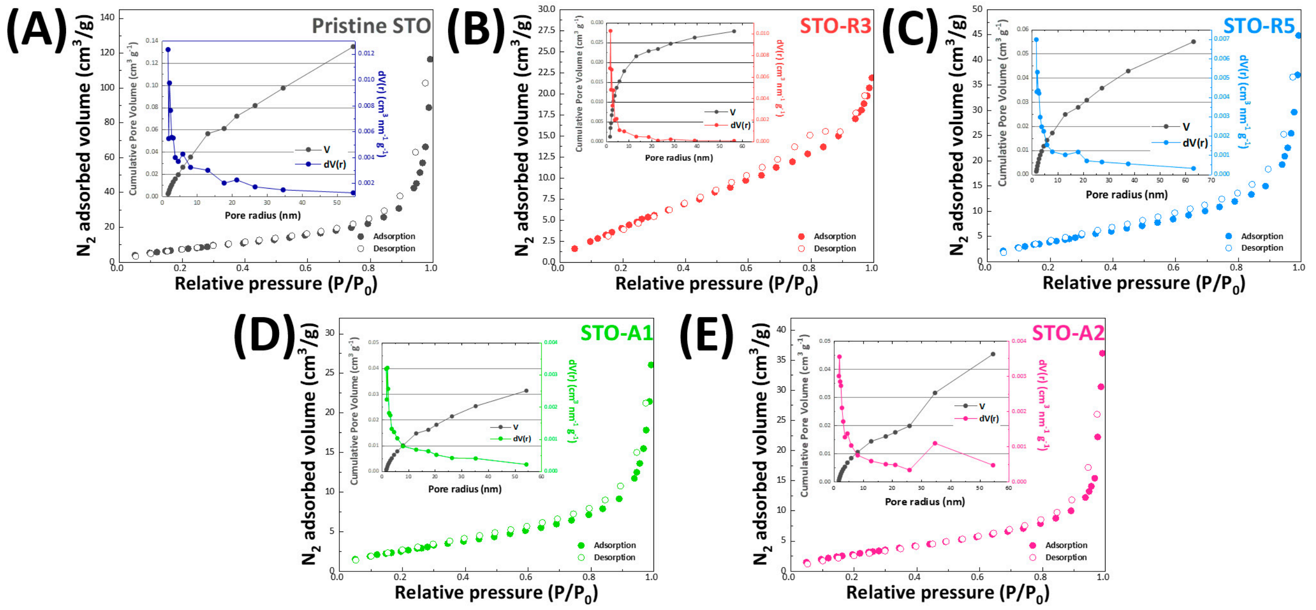

Figure S1. (**A-E**) N₂ absorption-desorption isotherms of Pristine-STO, STO-R3, STO-R5, STO-A1 and STO-A2 perovskites synthesized using the A-FSP process. Inset: Pore size distribution plot using the BJH method.

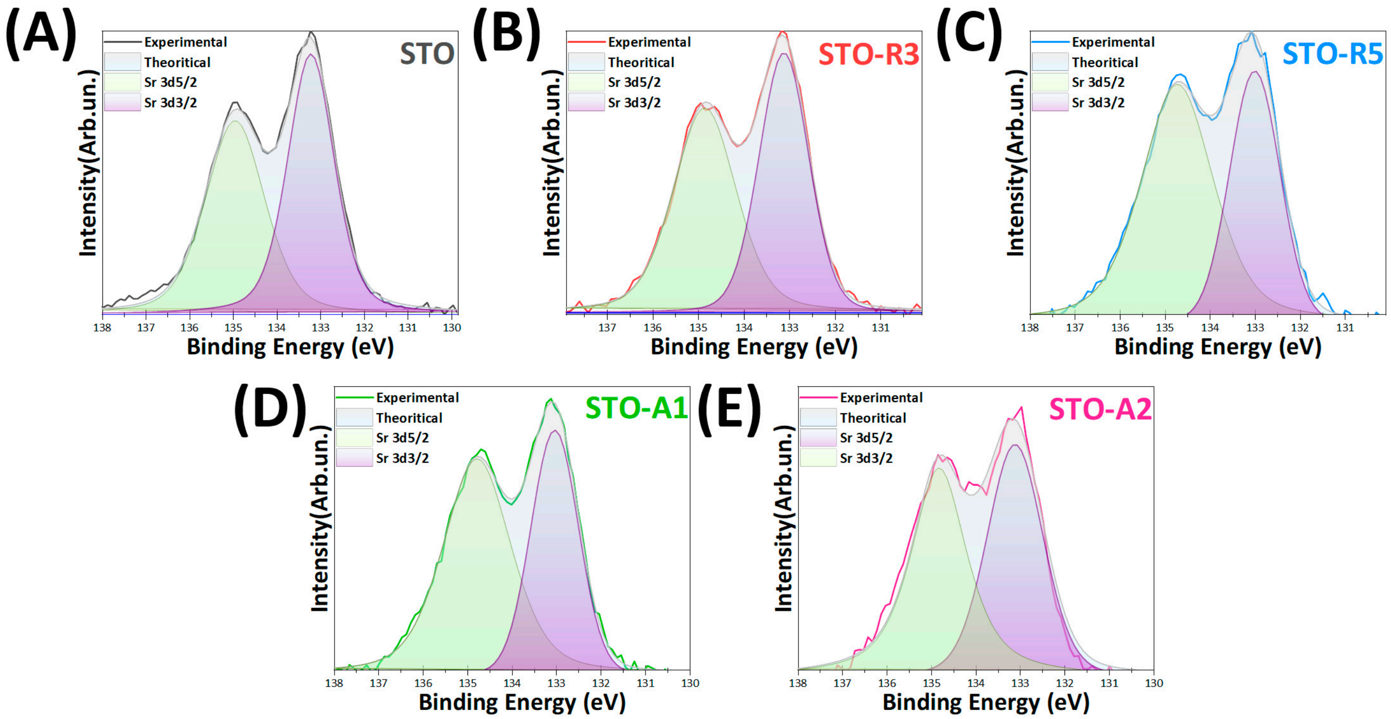

Figure S2. (A-E) Sr 3d XPS spectra of the five FSP and A-FSP-made STO nanomaterials.

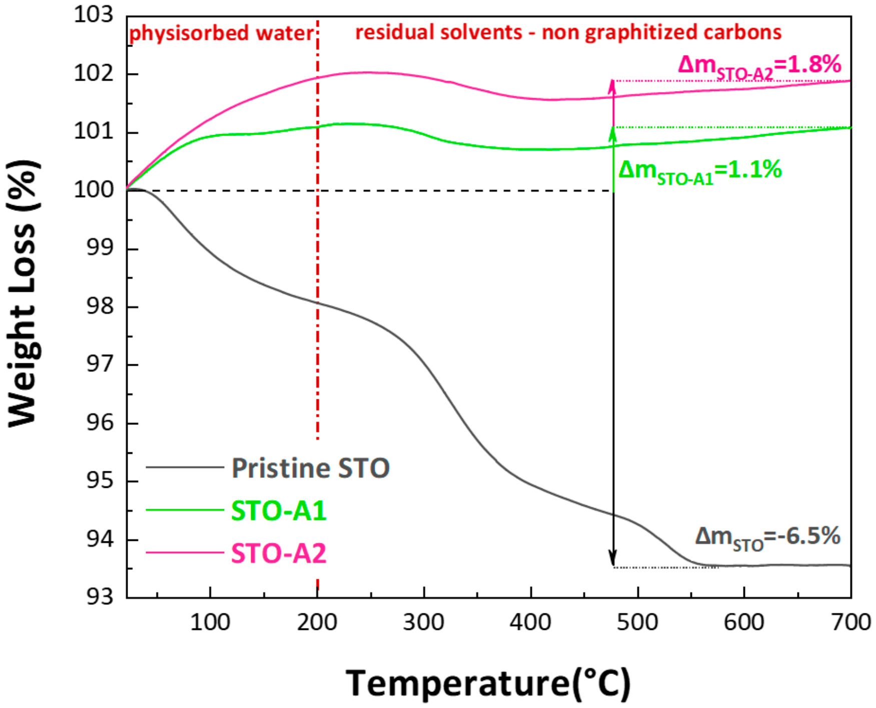

Figure S3. TGA data of the materials Pristine STO, STO-A1 and STO-A2 under synthetic air. Vertical arrows were used to mark the estimated mass change (Δm)
